# Mangiferin enhances the antifungal activities of caspofungin by destroying polyamine accumulation

**DOI:** 10.1080/21505594.2020.1870079

**Published:** 2021-01-06

**Authors:** Juan Shen, RenYi Lu, Qing Cai, LingZhi Fan, WanNian Yan, ZhenYu Zhu, LianJuan Yang, YingYing Cao

**Affiliations:** a Shanghai Skin Disease Hospital, Tongji University School of Medicine, Shanghai, P.R. China; b School of Pharmacy, Second Military Medical University, Shanghai, P.R. China

**Keywords:** Caspofungin, mangiferin, *C. albicans*, polyamines, ROS, SPE1

## Abstract

The incidence of fungal infections has increased continuously in recent years. Caspofungin (CAS) is one of the first-line drugs for the treatment of systemic fungal infection. However, the emerging CAS-resistant clinical isolates and high economic cost for CAS administration hamper the wide application of this drug. Thus, the combined administration of CAS with other compounds that can enhance the antifungal activity and reduce the dose of CAS has gained more and more attention. In this study, we investigated the effect of mangiferin (MG) on the antifungal activities of CAS. Our results showed that MG acted synergistically with CAS against various *Candida spp*., including CAS-resistant *C. albicans*. Moreover, MG could enhance the activity of CAS against biofilm. The *in vivo* synergism of MG and CAS was further confirmed in a mouse model of disseminated candidiasis. To explore the mechanisms, we found that SPE1-mediated polyamine biosynthesis pathway was involved in the fungal cell stress to caspofungin. Treatment of CAS alone could stimulate SPE1 expression and accumulation of polyamines, while combined treatment of MG and CAS inhibited SPE1 expression and destroyed polyamine accumulation, which might contribute to increased oxidative damage and cell death. These results provided a promising strategy for high efficient antifungal therapies and revealed novel mechanisms for CAS resistance.

## Introduction

In recent years, the incidence of fungal infections has become one of the major causes of morbidity and mortality of patients with serious underlying diseases, especially those hospitalized in intensive care units or undergoing treatment of hematological malignancies [1]. The leading *Candida* species, *Candida albicans*, is the most common pathogenic fungus that can cause different infections, ranging from cutaneous diseases to life-threatening disseminated candidiasis, especially in immunocompromised patients [2,3]. Nowadays, the main antifungal families include azoles, polyenes and echinocandins. Despite the availability of antifungal drugs, treatment of fungal infections is complicated by several limitations, such as off-target toxicity, high cost, and ineffectiveness against drug-resistant strains in patients undergoing treatment [4].

Caspofungin (CAS), which belongs to the echinocandin family, is one of the antifungal drugs frequently used for the treatment of fungal infection due to its low toxicity and high efficacy. CAS targets the fungal cell wall through inhibition of β-1,3-D-glucan synthase, an enzyme that synthesizes a major constituent of the fungal cell wall [5]. In addition, CAS can stimulate intracellular reactive oxygen species (ROS) production and cause oxidative damage, which is considered to be another important mechanism for its fungicidal activity [6]. CAS is effective in the treatment of fungal infections caused by *Candida albicans* and other *Candida spp*. However, with the number of patients exposed to CAS increasing rapidly, CAS-resistant clinical isolates emerge continuously [7–9]. In addition, the economic burden for the patients with CAS administration is much higher as compared to several other commonly used antifungals such as fluconazole. Thus, the combined treatment of CAS with other compounds that could potentially enhance CAS efficiency through exerting antifungal activities against CAS-resistant fungi and reducing the dose used for CAS is a promising strategy for the therapy of fungal infection [10,11].

**Figure 1.**
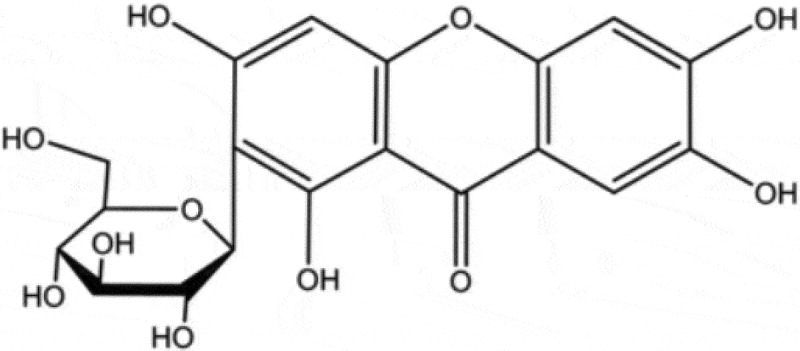
Structure of mangiferin

Nowadays, traditional Chinese medicines (TCMs) have attracted more and more attention due to their easy access, diverse bioactivities and lower toxicity. A series of compounds isolated from TCMs, such as shikonin, tetrandrine and baicalein, display excellent antifungal activities when used alone or combined with other antifungal agents [12,13]. Mangiferin (MG, 1,3,6,7-tetrahydroxy-C_2_-β-D-glucoside, Figure 1) is a xanthone and can be extracted from mango fruit as well as many other plants. MG has been reported to possess several health endorsing properties, including antioxidant, antiviral, anticancer, antidiabetic, antiallergic, and immunomodulatory [14]. In this study, we assessed the effect of MG on the antifungal activities of CAS and explored the potential mechanisms for the interaction of MG and CAS.

## Materials and methods

### Strains, cultures, and chemicals

The *C. albicans* reference strain SC5314 was kindly provided by professor Dominique Sanglard (Centre Hospitalier Universitaire Vaudois, Lausanne, Switzerland). The caspofungin-resistant *C. albicans* strains S20, S33, S36 were gifts from professor David S. Perlin (International Center for Public Health, Newark). The *Candida spp*. clinical isolates (including *C. albicans, C. parapsilosis* and *C. krusei*) were obtained from Shanghai Changhai Hospital and Skin Disease Hospital (Shanghai, China). The SPE1 null mutant (*spe1Δ/Δ*) and reintroduced strains were constructed in our previous study [15]. Stock cultures of the strains were routinely maintained on Sabouraud dextrose agar (4% w/v dextrose, 1% w/v peptone and 1.8% w/v agar) and grown in YPD liquid medium (1% yeast extract, 2% peptone, and 2% dextrose) with constant shaking at 30°C. MG and CAS were purchased from Sigma-Aldrich Co. LLC (St. Louis, MO, US). For all the experiments, 4 mg/ml MG dissolved in dimethyl sulfoxide (DMSO) and 1 mg/ml CAS dissolved in ultra-pure distilled water were used as stocks.

### Checkerboard test

The 96-well culture plates (Corning Inc., Corning, NY) were used for the checkerboard test according to a broth microdilution protocol of the Clinical and Laboratory Standards Institute M27-A3 method with some modification [16]. The initial concentration of fungal cells in RPMI 1640 medium (Sigma) buffered with 2% 3-(N-morpholino) propane sulfonic acid (MOPS) was 10^3^ CFU/ml. Both MG (concentration range, 0.25–128 μg/ml) and CAS (concentration range, 0.0313–16 μg/ml) were added in a 2-fold dilution. Plates were incubated at 35°C for 24 h. The minimum inhibitory concentration (MIC_80_) value was determined as the lowest concentration of the compounds tested that inhibited the growth of the fungal cells by 80%. The fractional inhibitory concentration (FIC) index is defined as the sum of the MIC_80_ of MG and CAS when used in combination divided by the MIC_80_ of MG or CAS used alone. Synergism and antagonism were defined by FIC indices of ≦ 0.5 and >4, respectively. The FIC indices of >0.5 but ≦ 4 was defined as indifferent.

### Time-killing assay

For time-killing assay, the *C. albicans* cells were cultured in an orbital shaker overnight for about 16 h, then transferred to a fresh YPD medium by 1:100 and continually grew for 6 h. Next, the cells were washed with phosphate-buffered saline (PBS) twice, resuspended in RPMI 1640 medium and adjusted to the concentration of 1 × 10^3^ cells/ml. Then, the cell suspensions were treated with drugs. For *C. albicans* SC5314, 8 μg/ml MG, 0.125 μg/ml CAS alone or in combination were added. For *C. albicans* CAS-resistant strain S20, 8 μg/ml MG, 0. 25 μg/ml CAS alone or in combination were added. The samples were further cultured with agitation at 30°C for 12 h. The cell suspensions were collected at different time points (3, 6, 9, 12 h), and plated on YPD agar to determine the CFU/ml of the cell suspensions.

### Biofilm growth and XTT reduction assay

The *in vitro* biofilm growth was performed according to the reference [17]. A batch of medium was inoculated from YPD agar plates containing freshly grown *C. albicans* cells and incubated overnight in an orbital shaker at 30°C. The cells were then suspended in RPMI 1640 medium and adjusted to the desired cell density (5 × 10^5^ cells/ml). The cell suspension was added to a 96-well tissue culture plate. Following the initial 90 min of adhesion at 37°C, the medium was aspirated, non-adherent cells were removed and fresh medium was added to the adherent cells to allow biofilm formation. The plate was further incubated at 37°C for 24 h. To determine the effect of the drugs on biofilm formation, fresh RPMI 1640 medium containing MG (16 μg/ml), CAS (0.25, 0.5 μg/ml) alone or in combination was added after 90 min of cell adhesion. To detect the effect of the drugs on mature biofilm, *C. albicans* biofilm was formed at 37°C for 24 h as described above, washed twice with PBS, then fresh RPMI 1640 medium containing MG, CAS alone or in combination was added. The plates were incubated at 37°C for a further 24 h.

The effect of the compounds on *C. albicans* biofilm growth was determined by a 2,3-bis- (2-methoxy-4-nitro- 5-sulfophenyl)-2 H-tetrazolium-5-carboxanilide (XTT) reduction assay [18]. Biofilm cells were washed twice with PBS and then incubated with 0.5 mg/ml of XTT and 1 mM of menadione in PBS at 37°C for 90 min. Then, a microtitre plate reader was used for the measurement of the optical density (OD) at 490 nm (A_490_).

### Measurement of *in vivo* antifungal activity

A mouse model of disseminated candidiasis was used for the evaluation of the antifungal activity *in vivo*. The animal experiments were approved by the Animal Ethics Committee of the Second Military Medical University (Shanghai, China). Six-week-old female BALB/c mice (Sino-British SIPPR/BK Lab Animal, Shanghai, China) were injected with 200 μl of *C. albicans* SC5314 cell suspension (5 × 10^6^ cells/ml in sterile saline) intravenously (0 day). MG (0.5 mg/kg) and CAS (0.03 mg/kg) was administered alone or in combination intraperitoneally on days 0, 1, 2, 4 and 6 after inoculation. Sterile saline solution was used as placebo. Everyday the mice were monitored for survival or sacrificed at day 3 post-infection for fungal burden assessment. For fungal burden assay, the kidneys were aseptically extracted, homogenized in 0.5 ml of PBS, and the log reduction in CFU/mg kidney tissue was calculated.

### Real-time RT-PCR

To investigate the mRNA expression of SPE1, DUR3, and FLU1 upon drug treatment, real-time RT-PCR experiment was conducted on a 7500 real-time PCR system (Applied Biosystems) as described previously [19]. *C. albicans* SC5314 cells were treated with 8 μg/ml MG, 0.125 μg/ml CAS alone or in combination for 4 h. The primers for SPE1, DUR3, and FLU1 are listed in Table S1 in the supplemental material. The fungal RNAout kit (TIANZ, Beijing, China) was used for total RNA isolation. The cDNA was acquired with the cDNA Synthesis Kit for RT-PCR (TaKaRa Biotechnology, Dalian, China). The amplified products in real time were monitored with SYBR green I (TaKaRa Biotechnology, Dalian, China). The expression of the genes was normalized to that of 18S rRNA.

### Reactive oxygen species (ROS) measurement

The production of ROS was measured with DCFH-DA (Molecular Probes, USA) as previously described [20]. Briefly, exponentially growing *C. albicans* cells were collected by centrifugation, washed twice with PBS and adjusted to 1 × 10^7^ cells/ml. Then, the cells were treated with 20 μg/ml of DCFH-DA and incubated with shaking at 30°C for 30 min. After washing the cells with PBS for three times, 8 μg/ml MG, 0.125 μg/ml CAS alone or in combination were added. To test the impact of polyamines on ROS production, putrescine, spermidine, and spermine were all added at the concentration of 2.5 mM. The cell suspensions were cultured with shaking at 30°C. At each designated time points, portions of the cell samples were harvested and 100 μl of the supernatant was transferred to the wells of a flat-bottomed 96-well microplate to detect fluorescence intensity using a fluorescence spectrometer (POLARstarGalaxy; BMG Labtech, Offenburg, Germany) with an excitation wavelength at 488 nm and an emission wavelength at 525 nm.

### Polyamines determination

In *C. albicans*, the main intracellular polyamines include putrescine, spermidine, and spermine. In this study, the effect of the compounds on polyamine accumulation was determined as described previously [15]. Briefly, *C. albicans* cells exposed to 8 μg/ml MG, 0.125 μg/ml CAS alone or in combination for 4 h were washed twice with PBS, resuspended in 5% perchloric acid, then lysed by sonication. The supernatant containing polyamines was collected. For benzoylation, 1 ml of 1 M NaOH solution and 50 μl of benzoyl chloride were added to 1 ml of the extracted solution. The mixture was shaken with a vortex mixer for 5 minutes and kept for 10 minutes at 37°C. After adding 2 ml of saturated sodium chloride and shaking for 1 min, the resulting derivatives were extracted twice with 1 ml aliquots of diethyl ether. The upper organic phases were evaporated to dryness, dissolved in methanol, and filtered for HPLC determination. HPLC was conducted using an Agilent 1100 Binary pump equipped with an Agilent 1100 dual wavelength UV−vis detector and a Shiseido C18 column (3.0 mm × 100 mm, 3 μm). The detector was set to 254 nm.

### Statistical Criteria

All experiments were performed at least three times independently. The minimum inhibitory concentration (MIC) data of the antifungal compounds were shown as 80% inhibition (MIC_80_) of the strains tested. Other data were calculated in GraphPad Prism 6.0 (GraphPad Software, San Diego, CA, United States) and expressed as the mean ± standard deviation. The significance of the difference was assessed using Student’s t-test. A P-value of <0.05, <0.01, or <0.001 was considered statistically significant.

## Results

### MG enhances the antifungal activities of CAS

The effect of MG on the antifungal activities of CAS was investigated by determining the lowest drug concentrations that displayed 80% growth inhibition (MIC_80_). As shown in Table 1, when used alone, CAS exhibited a much stronger antifungal activity than that of MG. For example, when used alone, the MIC_80_ values of CAS and MG against *C. albicans* reference strain SC5314 were 0.25 μg/ml and over 64 μg/ml, respectively. Combined treatment on SC5314 resulted in a markedly decrease in the MIC_80_ values of both CAS and MG, with the MIC_80_ values being 0.0625 μg/ml and 4 μg/ml, respectively. The corresponding FIC index was 0.313, indicating synergism of these two compounds. The synergistic interaction was observed not only in *C. albicans* clinical isolates and the reference strain SC5314, but also in other *Candida spp*., including *C. parapsilosis* and *C. krusei* clinical isolates, which are natively less sensitive to CAS as compared to *C. albicans*. The corresponding FIC index ranged from 0.094 to 0.375.

**Table 1.** Interaction of mangiferin and caspofungin against fungi

Strains	MIC_80_ alone^a^		MIC_80_ in combination		FIC index^b^
	MG	CAS	MG	CAS	
*Candida albicans* (CAS-S)					
SC5314	>64	0.25	4	0.0625	0.313
178	>64	0.25	4	0.0625	0.313
678	>64	0.5	2	0.0313	0.094
181	>64	0.5	2	0.0625	0.156
182	>64	0.25	4	0.0313	0.188
503	>64	0.25	4	0.0313	0.188
531	>64	0.25	4	0.0313	0.188
*Candida albicans* (CAS-R)					
S20	>64	8	4	0.25	0.094
S33	>64	8	4	0.25	0.094
S36	>64	8	4	0.25	0.094
*Candida parapsilosis*					
311	>64	1	2	0.125	0.156
327	>64	2	4	0.125	0.125
328	>64	1	4	0.125	0.188
*Candida krusei*					
213	>64	1	8	0.25	0.375
204	>64	0.5	4	0.125	0.313
266	>64	1	4	0.125	0.188

^a^The unit of mangiferin (MG) and caspofungin (CAS) is μg/ml.

^b^Synergism and antagonism were defined by FIC indices of ≦0.5 and >4, respectively. The FIC indices of >0.5 but ≦4 was defined as indifferent.

**Figure 2.**
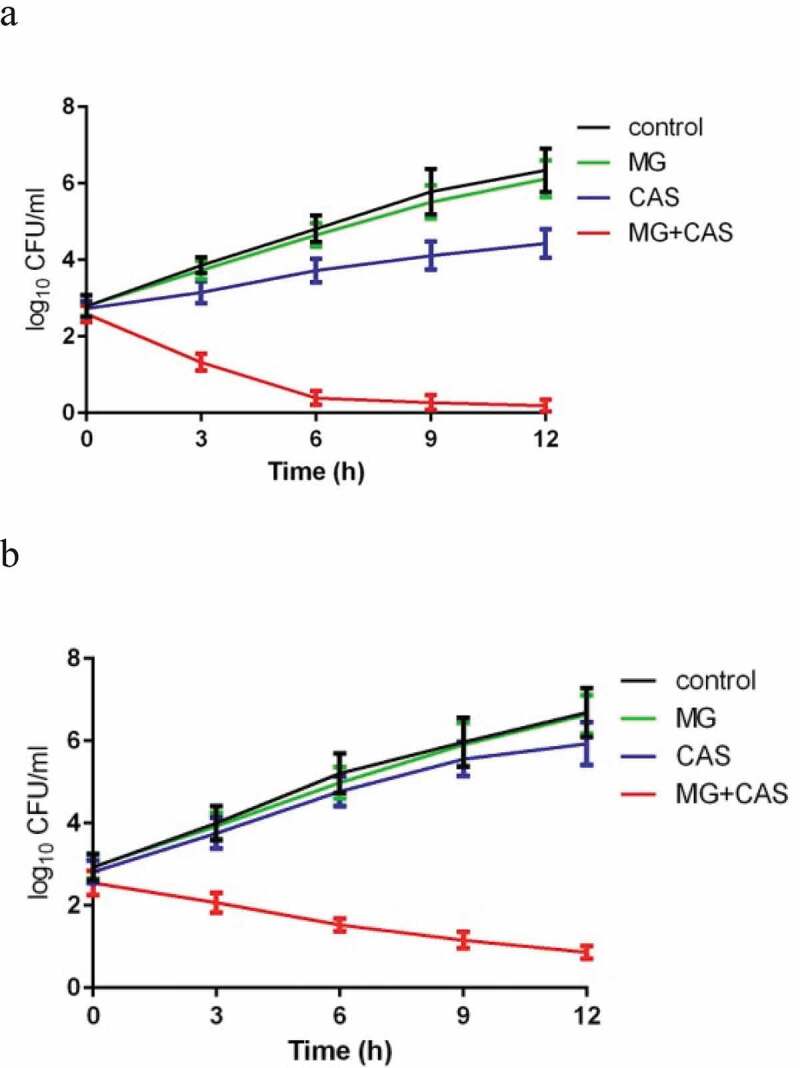
Time-kill curves of MG and CAS against *C. albicans*. (a) *C. albicans* SC5314 cells (1 × 10^3^ cells/ml) suspended in RPMI 1640 medium were treated by 8 μg/ml MG and 0.125 μg/ml CAS alone or in combination. At each designated time points, portions of the cell suspensions were collected and plated on YPD agar to calculate the CFU/ml of the cell suspensions. (b) *C. albicans* CAS-resistant strain S20 cells (1 × 10^3^ cells/ml) suspended in RPMI 1640 medium were treated by 8 μg/ml MG and 0.25 μg/ml CAS alone or in combination. At each designated time points, the cell suspensions were collected and plated on YPD agar to calculate the CFU/ml of the cell suspensions. Data were shown as the mean ± standard deviation of the independent assays in triplicate

The enhancement of the antifungal activity of CAS by MG was further confirmed by a time-killing test. As shown in Figure 2(a), when the *C. albicans* cells cultured with constant shaking were exposed to 8 μg/ml MG alone, no significant growth inhibition was observed. Exposure of the cells to 0.125 μg/ml CAS alone exhibited a slight growth inhibition. However, combined treatment of 8 μg/ml MG and 0.125 μg/ml CAS showed a strong antifungal activity, with the time-killing curve being remarkably dropped. After 12 h of treatment, approximately 4 log10 CFU/ml reduction was achieved in the combination group as compared to the CAS alone group.

### Synergism of MG and CAS against CAS-resistant strains

Since the rising number of CAS-resistant *C. albicans* strains occurring in hospitals is of concern and is often linked to the mutations in FKS1, which encodes the β-1,3-D-glucan synthase, we evaluated the interaction of MG and CAS on CAS-resistant *C. albicans* strains S20, S33, and S36, which contained various FKS1 mutations [21]. As shown in Table 1, the synergism of MG and CAS against CAS-resistant strains was similar to that against CAS-sensitive strains. The enhanced antifungal activity of CAS by MG was observed in all of the three CAS-resistant strains tested, reducing the MIC_80_ of CAS by 6 fold (from 8 μg/ml alone to 0.25 μg/ml in combination). Consistent with this, the time-killing curves showed that the combination of MG and CAS resulted in a much stronger antifungal activity than either CAS or MG treated alone (Figure 2(b)).

### MG enhances the activity of CAS against *C.albicans* biofilm

**Figure 3.**
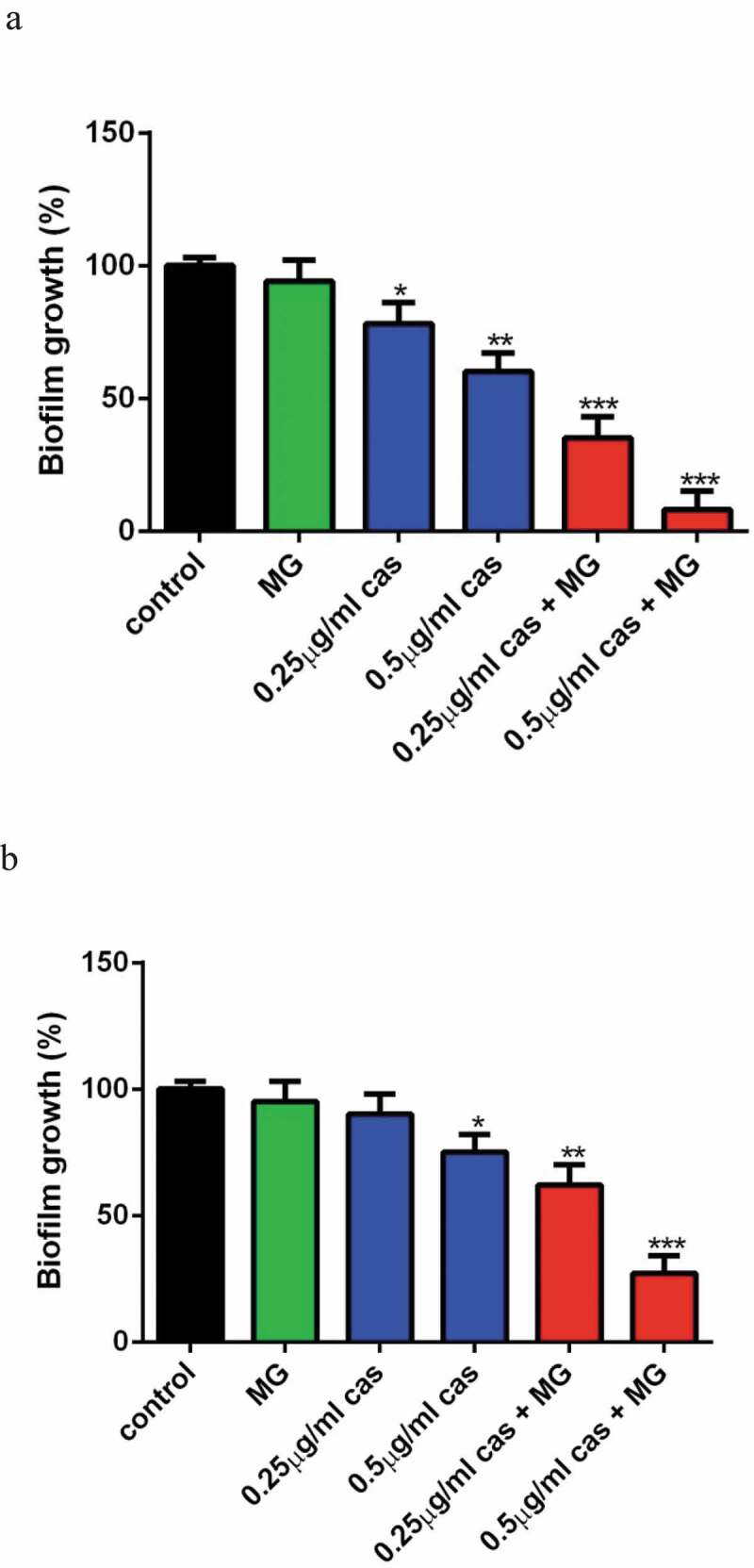
Interaction of MG and CAS against *C.albicans* biofilm. The biofilm growth of *C. albicans* SC5314 cells was determined by XTT reduction assay. (a) Effects of MG (16 μg/ml) and CAS (0.25, 0.5 μg/ml) alone or in combination on biofilm formation. MG and CAS alone or in combination was added after 90 min of cell adhesion. The biofilm cells were determined after incubated at 37°C for 24 h. (b) Effects of MG (16 μg/ml) and CAS (0.25, 0.5 μg/ml) alone or in combination on mature biofilms. *C. albicans* biofilm formed at 37°C for 24 h was washed twice with PBS, then fresh RPMI 1640 medium containing MG, CAS alone or in combination was added. The biofilm cells were were determined after incubated at 37°C for a further 24 h. Data were shown as the mean ± standard deviation of the independent assays in triplicate. *, *P*< 0.05; **, *P*< 0.01; ***, *P*< 0.001 as compared to the control group

Fungi biofilms show unique phenotypic traits and are less sensitive to CAS as compared to the planktonic cells, so we investigated the effect of MG on the activity of CAS against *C. albicans* biofilm. XTT reduction assay revealed that 16 μg/ml MG alone showed no significant effect on biofilm formation as compared to the control group. CAS at the concentration of 0.25 μg/ml alone showed a slight inhibitory activity on biofilm formation (P < 0.05). Combined treatment of 16 μg/ml MG and 0.25 μg/ml CAS led to a remarkable inhibition of biofilm formation (P < 0.001). An even severe inhibition of biofilm formation was observed when 16 μg/ml MG and 0.5 μg/ml CAS were used in combination (Figure 3(a)). The interaction of MG and CAS against mature *C.albicans* biofilm is shown in Figure 3(b). Although the growth of mature biofilm was hardly inhibited by 0.25 μg/ml CAS alone, about 40% of the mature biofilm was destroyed upon the combined treatment of 16 μg/ml MG and 0.25 μg/ml CAS. When 16 μg/ml MG and 0.5 μg/ml CAS were added in combination, the mature biofilm was destroyed by 73%. These results indicated that MG could strongly enhance the antibiofilm activity of CAS.

### MG enhances the antifungal activity of CAS *in vivo*

**Figure 4.**
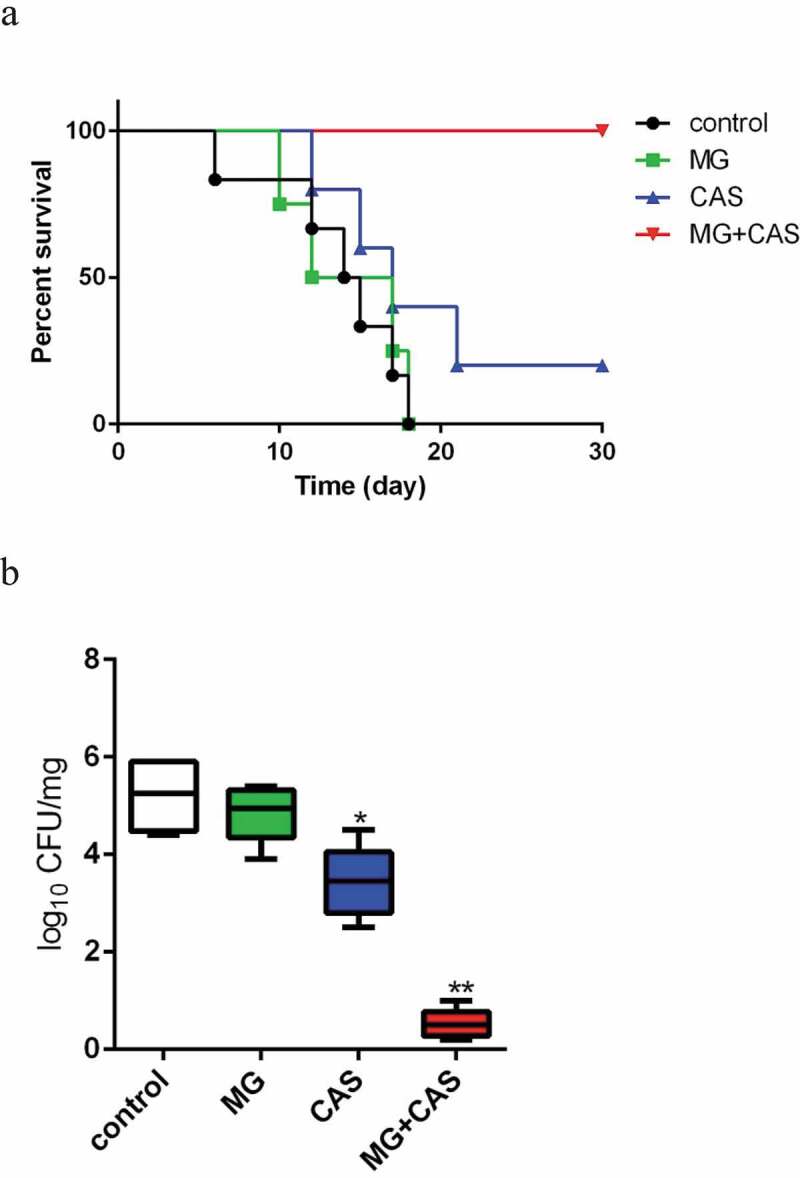
(a) Interaction of MG and CAS on the survival of mice with disseminated *candidiasis*. The BALB/c mice were injected with 200 μl of *C. albicans* SC5314 cells suspension (5 × 10^6^ cells/ml in sterile saline) intravenously (0 day). MG (0.5 mg/kg) and CAS (0.03 mg/kg) was administered alone or in combination intraperitoneally on days 0, 1, 2, 4 and 6 after inoculation. (b) The mice were sacrificed at day 3 post-infection for fungal burden assessment. The kidneys were aseptically extracted, homogenized, and the log reduction in CFU/mg kidney tissue was calculated. *, *P*< 0.05; **, *P*< 0.01 as compared to the control group

The *in vivo* interaction of MG and CAS was evaluated with a mouse model of disseminated candidiasis. As shown in Figure 4(a), there were no significant differences in the mice survival between the MG (0.5 mg/kg) alone and control (saline-treated) groups. Administration of CAS (0.03 mg/kg) alone showed a slightly prolonged mice survival period as compared to the control group. However, when 0.5 mg/kg MG and 0.03 mg/kg CAS were administrated in combination, the mice were absolutely protected from disseminated candidiasis and the survival rate was 100%. Consistently, fungal burden assay showed that treatment of MG alone could not significantly reduce the fungal burden, while treatment of CAS alone resulted in a decrease in the fungal burden as compared to the control group. However, there was a dramatic drop of fungal burden in kidney when MG and CAS were administrated in combination, in which the fungal cells seemed to be wiped out (Figure 4(b)).

### Combination of MG and CAS leads to increased ROS production

**Figure 5.**
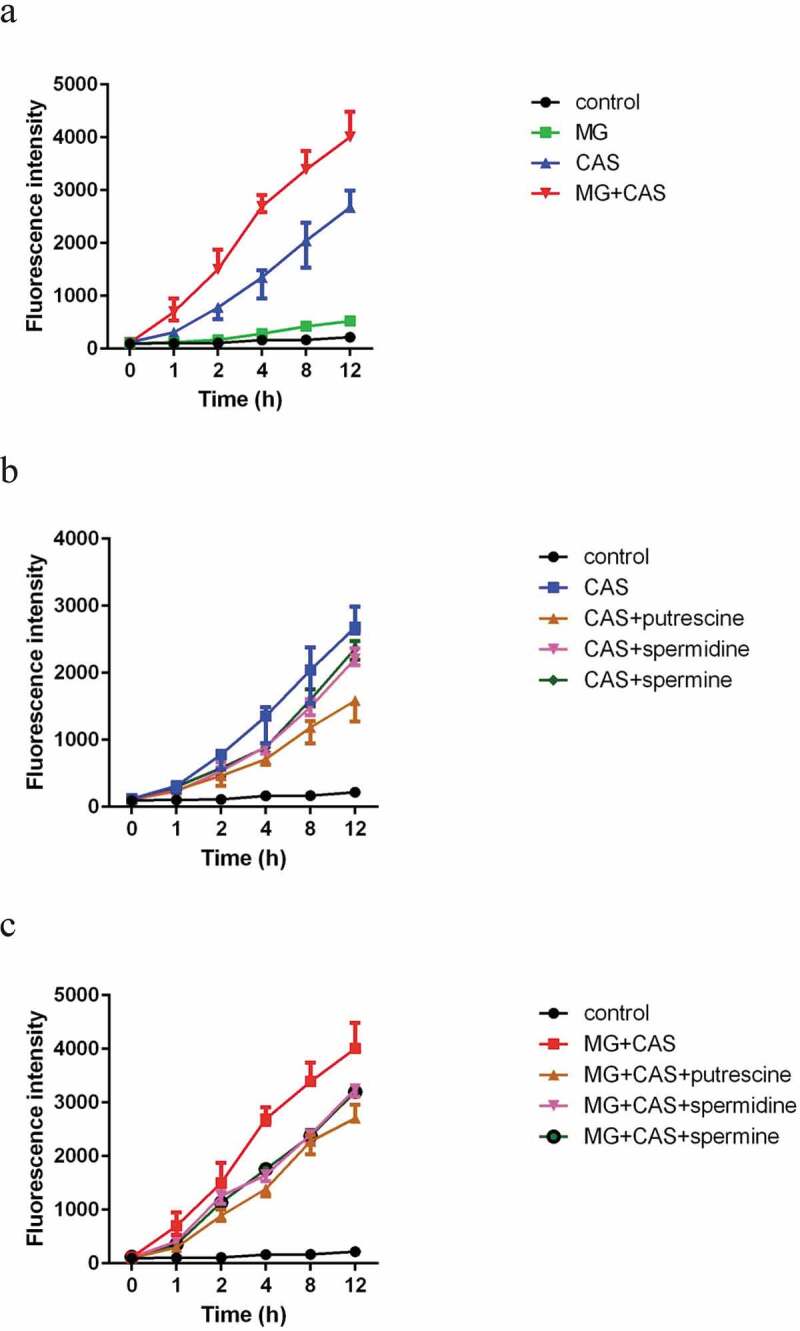
ROS production in *C. albicans* SC5314 cells. (a) The cells were exposed to 8 μg/ml MG, 0.125 μg/ml CAS alone or in combination. (b) The cells were exposed to 0.125 μg/ml CAS in the absence or presence of 2.5 mM polyamines (putrescine, spermidine, spermine). (c) The cells were exposed to the combination of 8 μg/ml MG and 0.125 μg/ml CAS in the absence or presence of 2.5 mM polyamines (putrescine, spermidine, spermine). At the indicated time points, portions of the cell samples were taken to measure the ROS level using a fluorescence spectrometer. Data were shown as the mean ± standard deviation of the independent assays in triplicate

Since the induction of intracellular ROS is one of the important mechanisms for the antifungal activity of CAS [6], we determined the intracellular ROS level with the fluorescent dye DCFH-DA. As shown in Figure 5(a), there were no significant differences in ROS production between the *C. albicans* cells treated by 8 μg/ml MG alone and control (untreated) groups. Addition of 0.125 μg/ml CAS alone significantly stimulated the production of ROS. Combined treatment of 8 μg/ml MG and 0.125 μg/ml CAS led to an even higher ROS level as compared to the 0.125 μg/ml CAS alone group. This result suggested that MG might potentiate the antifungal activity of CAS through increasing ROS-mediated oxidative damage.

### Combination of MG and CAS destroys polyamines accumulation

**Figure 6.**
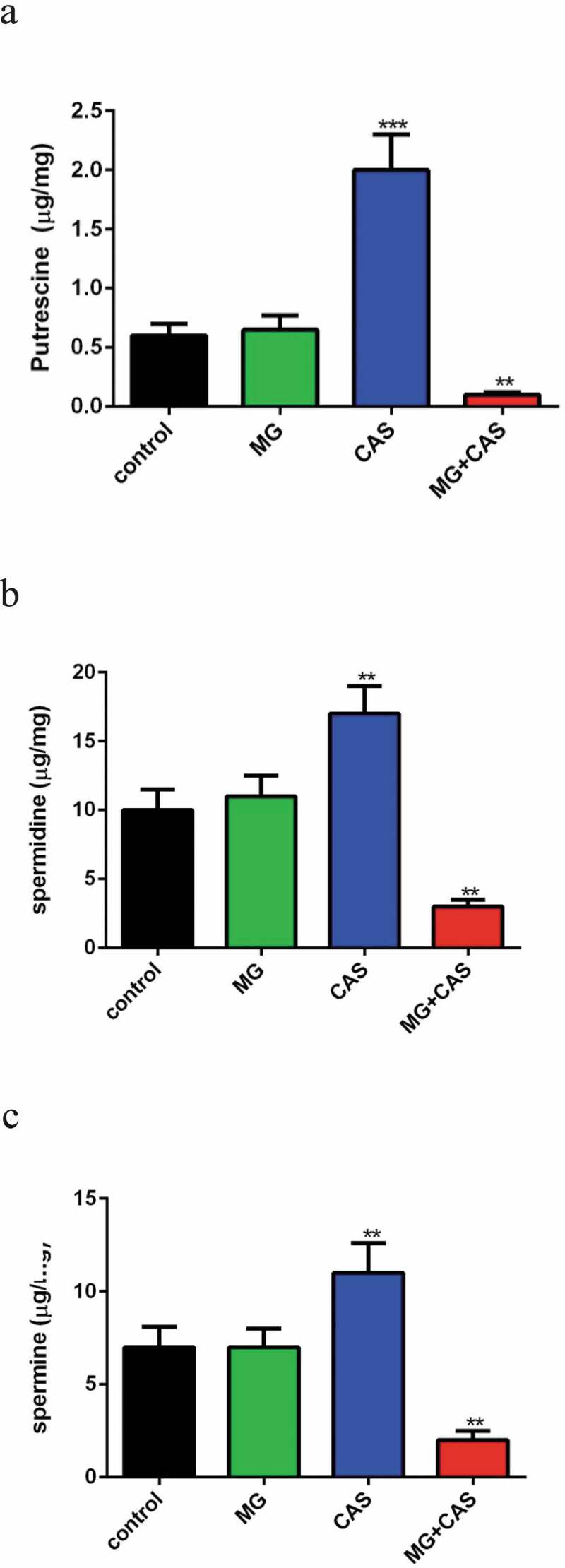
Measurement of polyamines contents. *C. albicans* SC5314 cells were exposed to 8 μg/ml MG, 0.125 μg/ml CAS alone or in combination for 4 h and the intracellular putrescine (a), spermidine (b) and spermine (c) were determined with HPLC. Data were shown as the mean ± standard deviation of the independent assays in triplicate. **, *P*< 0.01; ***, *P*< 0.001 as compared to the control group

Previous research pointed to a specific role of fungi polyamines in ROS scavenging and cellular protection against oxidative damage [15]. In view of the dramatic increase of ROS level upon combined treatment of MG and CAS, we determined the intracellular accumulation of polyamines. The levels of the polyamines in *C. albicans* cells treated by 8 μg/ml MG alone were similar to the untreated cells. Addition of 0.125 μg/ml CAS alone resulted in a remarkable accumulation of all of the polyamines tested, including putrescine (Figure 6(a)), spermidine (Figure 6(b)), and spermine (Figure 6(c)), among which putrescine accumulated the most. However, the accumulation of polyamines was destroyed severely when 8 μg/ml MG and 0.125 μg/ml CAS were added in combination, with the level of putrescine dropping by approximately twenty fold as compared to that of the CAS alone group.

### Exogenous addition of polyamine attenuates the antifungal activity of CAS

**Figure 7.**
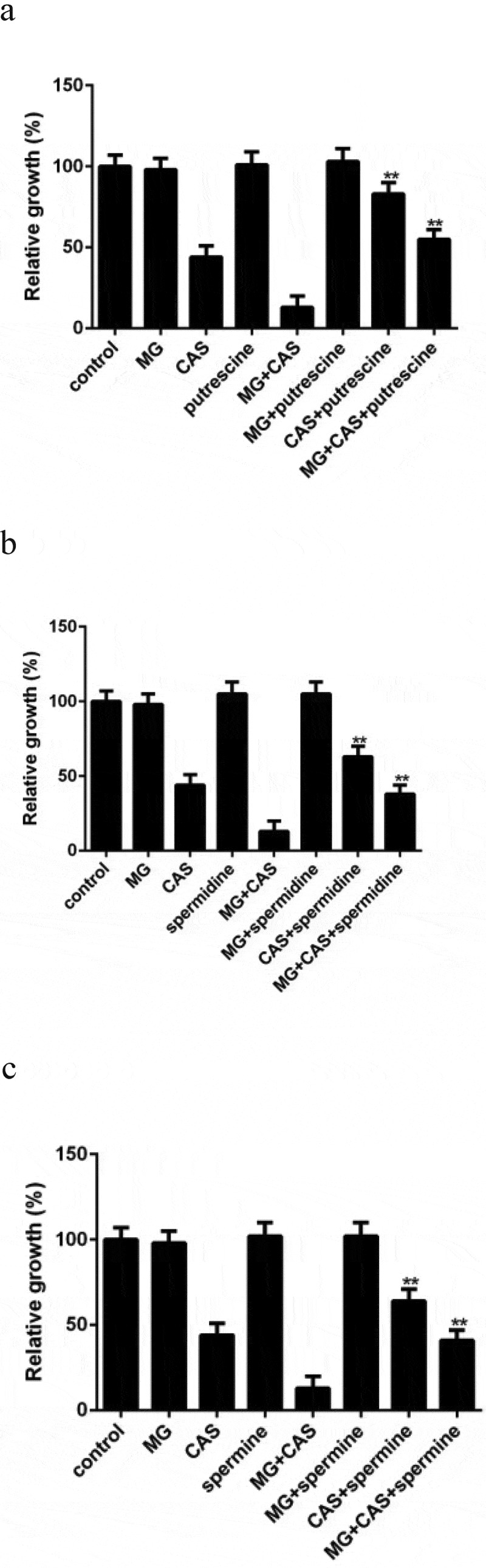
Effect of exogenous addition of polyamines on the antifungal activity of MG and CAS. *C. albicans* SC5314 cells which were exposed to 8 μg/ml MG and 0.125 μg/ml CAS alone or in combination were simultaneously treated with 2.5 mM Putrescine (a), spermidine (b) or spermine (c). The OD values were recorded after 6 h of growth and were represented relative to the OD values obtained from the control group. Data were shown as the mean ± standard deviation of the independent assays in triplicate. **, *P*< 0.01 as compared to the corresponding polyamine-free group

Given the fact that the polyamine levels in *C. albicans* cells were changed upon drug treatment, we investigated the role of polyamines in drug sensitivity through exogenous addition of polyamines. None of the polyamines affected the growth of the *C. albicans* cells exposed to MG alone. Addition of putrescine could significantly enhance the growth of the cells exposed to CAS alone. This enhancement was also observed when putrescine was added to the combination group (Figure 7(a)). Similar results were obtained when the other two polyamines, spermidine and spermine, were added (Figure 7(b,c)). These results indicated that exogenous addition of polyamines could not only attenuate the antifungal activity of CAS when used alone but also attenuate the antifungal activity of CAS when combined with MG.

In view of the impact of polyamines on drug sensitivity, we speculated that polyamines might affect ROS production. Consistent with the attenuated antifungal activity of CAS caused by polyamines, all of the polyamines tested could reduce the ROS levels in the *C. albicans* cells stimulated by CAS. The decrease of ROS levels caused by exogenous addition of polyamines was observed in both the CAS alone (Figure 5(b)) and the combination groups (Figure 5(c)).

### Deletion of polyamines biosynthesis gene SPE1 results in high sensitivity to CAS

**Figure 8.**
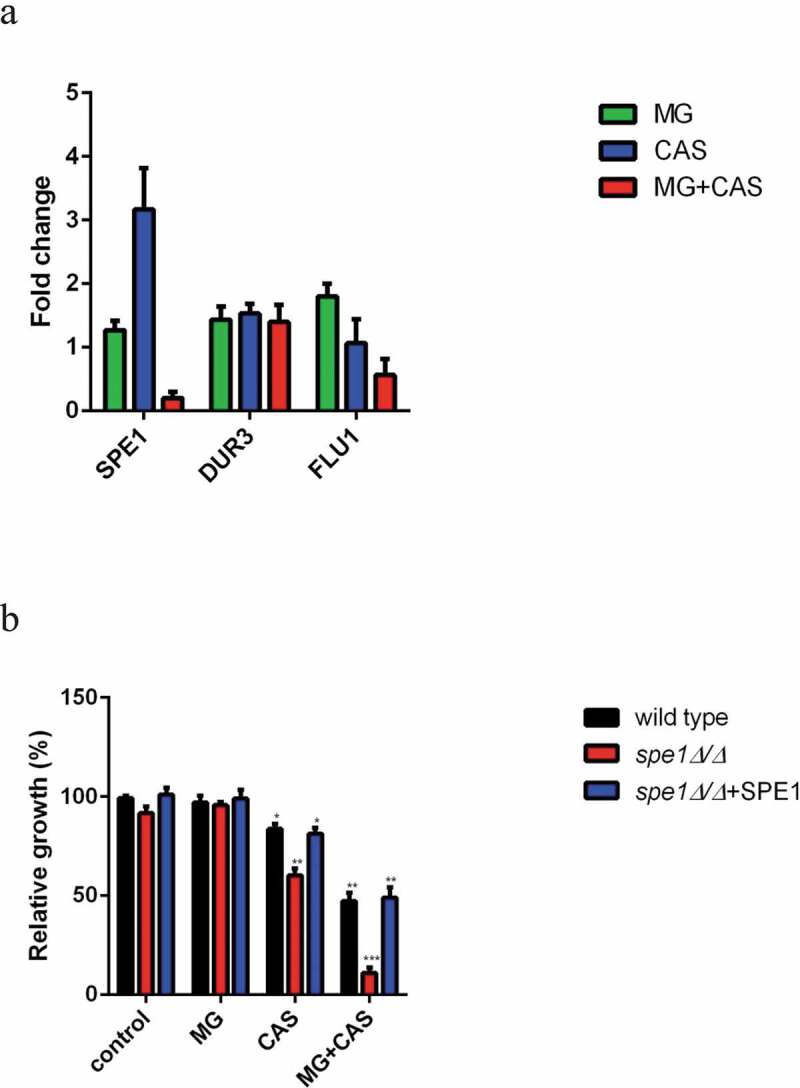
(a) The mRNA expression of SPE1, DUR3 and FLU1 in *C. albicans* SC5314 following treatment of 8 μg/ml MG and 0.125 μg/ml CAS alone or in combination for 4 h. The mRNA levels were normalized on the basis of their 18S rRNA levels. Gene expression was indicated as the fold increase in the drug-treated groups relative to that of the control group. (b) *C. albicans* wild type strain (SN152), *spe1Δ/Δ* mutant and SPE1 reintroduced (*spe1Δ/Δ*+ SPE1) strains were exposed to 8 μg/ml MG and 0.06 μg/ml CAS alone or in combination. The OD values were recorded after 6 h of growth and were represented relative to the OD values obtained from the control group. Data were shown as the mean ± standard deviation of the independent assays in triplicate. *, *P*< 0.05; **, *P*< 0.01; ***, *P*< 0.001 as compared to the control group

We next determined the mRNA expression of the *C. albicans* genes involved in polyamine accumulation including SPE1, which encodes an ornithine decarboxylase required for polyamine biosynthesis, polyamine intake gene DUR3, and efflux gene FLU1 [22–24]. As shown in Figure 8(a), MG alone did not affect the expression of SPE1 mRNA, while treatment of CAS alone resulted in a higher expression of SPE1 mRNA as compared to the control group. However, the expression of SPE1 mRNA was significantly inhibited when MG and CAS were added in combination. In addition, there was no significant change in the expression of DUR3 or FLU1 mRNA upon exposure of MG or CAS alone or in combination.

We next investigated the role of SPE1 in drug sensitivity through gene deletion of SPE1. As shown in 8B, the growth of *spe1Δ/Δ* mutant exposed to CAS alone was much lower than that of the wild type or SPE1 reintroduced strains, indicating high sensitivity of this mutant to CAS. Similarly, when this mutant was exposed to the combination of MG and CAS, a dramatic decrease in cell growth was observed as compared to the wild type or SPE1 reintroduced strains. Of note, deletion of SPE1 did not affect the growth of the cells exposed to MG alone.

## Discussion

Nowadays, pathogenic fungi have emerged as a significant cause of life-threatening infections and are often associated with a high mortality rate [25,26]. In spite of the availability of new drugs, the antifungal activities remain suboptimal in some cases and there is increasing evidence of antifungal therapy failing [27–29]. CAS is the first approved echinocandin and is used in the clinic for approximately 20 years. However, the corresponding higher economic burden associated with fungal therapy and the emergence of CAS-resistant *Candida* species hinder the wide application of CAS in the clinic. In this study, we found that MG, a bioactive natural compound widely existed in plants, could enhance the antifungal activity of CAS. Our results showed that MG acted synergistically with CAS against various *Candida spp*., including *C. albicans, Candida parapsilosis,* and *Candida krusei*. Moreover, the synergism was observed in CAS-resistant *C. albicans*. Further *in vivo* experiments revealed that combined administration of MG and CAS could absolutely protect the mice from disseminated candidiasis, while administration of CAS alone at the combination dose only displayed a slight protection effect.

The formation of biofilm is one of the important virulence factors of pathogenic fungi, which makes the fungi resistant to many commonly used antifungal agents, including CAS [30]. Here we investigated the effect of MG on the antibiofilm activity of CAS and our results revealed a remarkable enhancement of the activity of CAS against biofilm in the presence of MG, while MG alone did not exhibit antibiofilm activity. These results suggested that the combination treatment of MG and CAS might be a promising strategy in overcoming various clinical fungal infections.

Polyamines are aliphatic polycations essential for cell growth and differentiation. In fungi, putrescine, spermidine, and spermine are three major polyamines, which act as cellular protectors against a variety of stress challenges [31,32]. In *C. albicans*, putrescine is produced through decarboxylation of ornithine. Putrescine is the precursor for the biosynthesis of spermidine and spermine and can be further processed into these two polyamines. In addition to the roles of polyamines in stabilizing cellular membranes and binding to nucleic acids, scavenging ROS, which are a variety of molecules that can cause oxidative damage to DNA, proteins, and lipids and eventually cell death, is an important function attributed to polyamines [33–35]. Previous research reported that, besides inhibiting cell wallβ-1,3-glucan synthesis, stimulating the production of intracellular ROS was another important mechanism for the antifungal activity of CAS [6]. In this study, high ROS level was detected in the *C. albicans* cells exposed to CAS alone, while combined treatment of CAS and MG resulted in an even higher ROS level. These results prompted us to determine the intracellular levels of polyamines. We detected high accumulation of putrescine, spermidine, and spermine in the *C. albicans* cells exposed to CAS alone. A previous metabolomics study revealed that accumulation of polyamines was a stress response of the *C. albicans* cells upon exposure to amphotericin B, which led to enhanced scavenging of intracellular ROS and thus enabled the cells to survive the antifungal effects [15]. So the high levels of putrescine, spermidine and spermine stimulated by CAS might also be a stress response that played an important role in protecting the *C. albicans* cells from CAS killing. Consistent with this, our further experiment showed that exogenous addition of polyamines could significantly attenuate the antifungal activities of CAS, which was accompanied with reduced ROS production.

It should be noted that the stress response of polyamine accumulation in *C. albicans* cells was specific to CAS alone, as the levels of the polyamines were decreased dramatically when MG and CAS were added in combination. We speculated that lack of intracellular polyamine accumulation might weaken the ability of *C. albicans* cells to scavenging ROS, which led to increased oxidative damage and cell death in the combination group. The demonstration that destroying polyamine pools might contribute to the synergistic interaction of MG and CAS could also be explained by the factor that the antifungal mechanism for the synergism was unrelated to FKS1 mediatedβ-1,3-glucan biosynthesis, as the synergistic interactions were observed not only on CAS-sensitive strains but also on CAS-resistant FKS1 mutants.

The fungal polyamines mainly include putrescine, spermidine, and spermine, and they are strictly adjusted to normal levels through self biosynthesis, intake, and efflux [36,37]. In *C. albicans*, the precursor of polyamine biosynthesis is ornithine. Ornithine can be catalyzed by ornithine decarboxylase (ODC), which is the key enzyme for polyamine synthesis and encoded by SPE1, to produces the primary polyamine putrescine. Putrescine is further processed into spermidine and spermine [22]. It was reported that the ODC activity could be altered in response to various extracellular stimuli such as hormones and growth factors, as well as changes in intracellular polyamines [38]. To explore the mechanisms for the changed levels of polyamines upon drug treatment, we determined the mRNA expression of SPE1, and two other genes, DUR3 and FLU1, which are responsible for polyamine intake and efflux, respectively [23,24]. Our results showed that there was no significant change in the expression of DUR3 or FLU1 upon drug treatment. However, the expression of SPE1 was up-regulated in the CAS alone group but down-regulated in the combination group. These results were consistent with the differences in the polyamine levels between the two groups, indicating that SPE1 might play an important role in accurately regulating the polyamine pool in response to the antifungal stress. When the *C. albicans* cells were exposed to CAS alone, the expression of SPE1 mRNA was increased, which resulted in enhanced ODC activity, polyamine accumulation, and the corresponding high ability to scavenging ROS. In contrast, exposure to the combination of CAS and MG led to down-regulation of SPE1 expression. Thus, the ODC activity was reduced, which was accompanied with decreased polyamine level and ability to scavenging ROS. In this study, the role of SPE1 in antifungal stress response was further confirmed by the test on *spe1Δ/Δ* mutant. Given the function of the ornithine decarboxylase encoded by SPE1 as the key and first rate limiting enzyme in polyamine synthesis, it was understandable that the *spe1Δ/Δ* mutant was more sensitive to CAS either in the absence or presence of MG as compared to the wild type strain.

Taken together, our results demonstrated that MG could enhance the antifungal activities of CAS both *in vitro* and *in vivo*. The synergistic interaction of MG and CAS was observed in not only CAS-sensitive *Candida spp*., but also in CAS-resistant strains as well as biofilms. This combination is a promising recipe for reducing the dose and enhanced the antifungal activity of CAS. Moreover, our study revealed that polyamine accumulation, which contributing to ROS scavenging, was an important stress response in the *C. albicans* cells upon exposure to CAS killing. Destroying the polyamine pools through the combined treatment of MG and CAS led to enhanced antifungal activity. These results revealed a novel mechanism for CAS resistance and shed new light on the strategy for developing high efficient antifungal therapies.
